# The Optimum Production Method for Quality Improvement of Recycled Aggregates Using Sulfuric Acid and the Abrasion Method

**DOI:** 10.3390/ijerph13080769

**Published:** 2016-07-29

**Authors:** Haseog Kim, Sangki Park, Hayong Kim

**Affiliations:** 1 Building and Urban Research Institute, Korea Institute of Civil Engineering and Building Technology, 283, Goyang-daero, Ilsanseo-gu, Goyang-si 10223, Korea; bravo3po@kict.re.kr; 2 Department of Civil & Environmental Engineering, Kongju National University, 1223-24, Cheonan-daero, Seobuk-gu, Cheonan-si 31080, Korea; civilkhy@kongju.ac.kr

**Keywords:** recycled fine aggregates, response surface methodology, design of experiment, orthogonal arrays, abrasion-crusher time, acid treatment

## Abstract

There has been increased deconstruction and demolition of reinforced concrete structures due to the aging of the structures and redevelopment of urban areas resulting in the generation of massive amounts of construction. The production volume of waste concrete is projected to increase rapidly over 100 million tons by 2020. However, due to the high cement paste content, recycled aggregates have low density and high absorption ratio. They are mostly used for land reclamation purposes with low added value instead of multiple approaches. This study was performed to determine an effective method to remove cement paste from recycled aggregates by using the abrasion and substituting the process water with acidic water. The aim of this study is to analyze the quality of the recycled fine aggregates produced by a complex method and investigate the optimum manufacturing conditions for recycled fine aggregates based on the design of experiment. The experimental parameters considered were water ratio, coarse aggregate ratio, and abrasion time and, as a result of the experiment, data concerning the properties of recycled sand were obtained. It was found that high-quality recycled fine aggregates can be obtained with 8.57 min of abrasion-crusher time and a recycled coarse aggregate ratio of over 1.5.

## 1. Introduction

There has been increased deconstruction and demolition of reinforced concrete structures due to the aging of the structures and redevelopment of urban areas, i.e., urban renewal, reindustrialization, and large-scale housing reconstruction plans, which result in the generation of massive amounts of construction waste that cause damage to the urban and residential environments [1,2]. There are more than 300 construction waste treatment companies in Korea with booming businesses that are producing recycled aggregates. However, the recycled aggregates manufactured by these companies are not being used from multiple approaches, but are instead used in low value-added fields, such as land reclamation. This is thought to be due to the low quality of the recycled aggregates. The volume of construction wastes generated has risen dramatically, exceeding 68 million tons in 2012, and it is expected to rise above 100 million tons by 2020 [3]. Accordingly, the production volume of waste concrete, which accounts for the highest proportion of construction wastes, increased to 42 million tons in 2012, and it is projected to increase to over 100 million tons by 2020 [4]. This has made it necessary to develop technologies for the recycling of waste concrete and establish the necessary measures for their practical use. Although there have been research and development efforts to derive a wide range of technologies and methods to produce high-quality recycled aggregates, the recycled aggregates that are manufactured using the current technology are incomparable natural aggregates in terms of quality, and this has resulted in limitations of their use. Thus, considering the aspect of effective utilization of resources, there is a need to promote the high-value-added use of recycled aggregates. Based on this, there is high value in research performed to enhance the quality of recycled aggregates.

**Figure 1:**
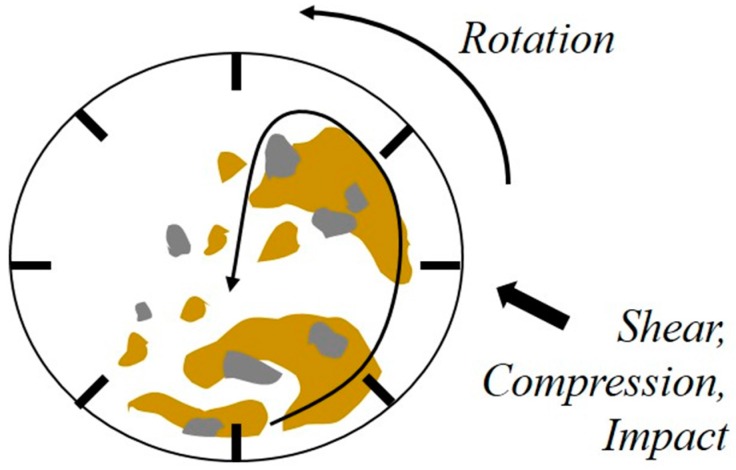
Principle of abrasion crusher.

The reason behind the low quality of recycled fine aggregates is that the cement paste attached to the aggregate surface has a negative impact on the absorption ratio and specific gravity [5]. Accordingly, there have been numerous studies conducted with the aim to effectively remove cement paste from aggregate surface. Most common methods of such removal are the mechanical method in which the cement paste is removed by physically crushing and grinding the waste concrete and the innovative method using heat or acid. Although a crushing process, for example, can lead to a significant reduction of the cement paste content, there are issues of increased costs and an overall reduction in the production volume of coarse aggregates as it involves the crushing of relatively weak aggregates into fine aggregates [6,7,8]. In order to resolve these issues, acid may be used as a means to remove only the cement paste, which is alkaline and can be removed through a neutralization reaction. It has been reported that the use of sulfuric and hydrochloric acids results in a high rate of cement paste removal [9,10,11]. Additionally, using a rotary mixing system in addition to the acid treatment can enhance the reactivity between the cement paste and the acidic substance, thereby facilitating the removal of the cement paste and alumino-silicate gel [12]. Thus, in this study, in addition to executing acid treatment, the abrasion method in combination with a chemical reaction was applied as shown in Figure 1. The mechanisms of abrasion can be largely divided into compression, impact, shearing, and friction. However, it is highly rare for only one of these abrasion mechanisms to occur; instead, generally speaking, two or more types of abrasion mechanisms occur in combination [5,7,13]. In order to improve the efficiency of abrasion, various forms of abrasion media may be combined in order to apply higher energy to the ground item and enhance the grinding efficiency [14,15].

Accordingly, this study was conducted with the aim to mitigate the issues arising from the crushing and grinding methods used in the conventional recycled aggregate production practices and to empirically and statistically review and analyze the optimum conditions in which recycled fine aggregates could be produced after neutralizing strong alkaline water. In other words, the amount of washing water, which exerts an impact during abrasion, the amount of coarse aggregates to be ground, and the abrasion time were chosen as the experimental parameters, and the effectiveness of the recycled fine aggregate and water ratio, the recycled fine aggregate, and coarse aggregate ratio, and the abrasion time were reviewed under an orthogonal design. Then, the optimum abrasion conditions for the production of recycled fine aggregates were derived using the response surface methodology (RSM). During the optimization procedure, unit production amount was expressed as a function of coarse aggregate ratio and the abrasion time and was taken as the objective function. Nelder-Mead sequential simplex algorithm, i.e., *fminsearch* function in MATLAB (R2015b, the Mathworks Inc., Natick, MA, USA, 2015), was applied to solve the problem with various constraints.

## 2. Experimental Design and Method

### 2.1. Experiment Design

In general, the orthogonal design method [16], proposed by Genichi Taguchi, is an experimental design method that can reduce a number of experiments by means of sacrificing the information on the parameters effecting on the test results. Therefore, the orthogonal design method has been adopted to estimate variable interactions among water ratio, coarse aggregate ratio, and abrasion time [17]. In this study, a three-level design system, mainly used in cases where factors are measured values, was used as represented in Equation (1), which is commonly used when the parameters are indiscrete values, was used [18]:

(1) $$L_{3m}=3^{\left(\frac{3m-1}{2}\right)}$$

where *L* is an orthogonal array; *m* is an integer number of experiment factor; 3*m* is a size of the experiment; and (3*m* − 1)/2 is the number of columns in the orthogonal array.

Additionally, RSM employed in order to derive the optimum abrasion method for the production of recycled fine aggregates is a statistical analysis method focusing on the response surface on which changes occur when multiple explanatory variables $(\xi_{1,}\xi_{2,}\xi_{3,\;}$…… $\xi_{n}$) exert an impact on a certain variable η in a complex manner [7]. RSM used in this study employed the secondary regression model, and the T-surface is expressed as shown in Equation (2):

(2) $$\eta =\beta_{0}+\sum^{\text{​}}\beta_{ij}\chi_{i}\chi_{j}$$

where η is an dependent variable; χ is an independent variable; and β is an constant.

As for the experimental design and level of this study, three factors, i.e., the fine aggregate to water ratio, the fine aggregate to coarse aggregate ratio, and abrasion time, were selected as the experimental parameters, as shown in Table 1 and 27 experimental batches were arranged in nine experimental levels, as shown in Table 2, using an orthogonal design based on the experimental design. It should be commented that the water ratio is the ratio of the volume of water to the volume of total aggregates. When the washing water ratio is less than 0.7, it is impossible to conduct experiments due to flocculation. If the washing water ratio exceeds 1.3, the grinding efficiency is decreased. Based on these reasons, two values, i.e., 0.7 and 1.3, are selected as a lower and an upper boundary, respectively. The coarse aggregate ratio is the ratio of the weight of coarse aggregates to the weight of fine aggregates. If the coarse aggregate ratio is under 0.5, then the grinding efficiency is decreased. On the contrary, if it is over 1.5, fine aggregates are ground with a size of 1.2 mm or less due to the excessive crushing action. Therefore, a lower and an upper boundary for the coarse aggregate ratio is 0.5 and 1.5, respectively.

In addition, based on the results of applying the orthogonal design, RSM was used to derive the optimum abrasion conditions. The coarse aggregates to be ground in this experiment were broken pebbles that were bigger than 100 mm, and they were to replace the fine aggregates in terms of weight. The amount of washing water to be used was determined for the total volume of fine and coarse aggregates. The abrasion time was divided into three levels: 5 min, 10 min, and 15 min. This was because field application of this experimental method would decrease the production rate for long-term processing and reduce the cost effectiveness.

### 2.2. Experimental Methods

The recycled fine aggregates, having a size of 5 mm or less, used in this experiment were obtained from Green and Environments Co., Cheonan, Korea and their physical properties are shown in Table 3. Additionally, the washing water used in this experiment was water from the common waterworks, which is typically used by recycled aggregate manufacturers.

It should be noted that the fineness modulus (FM) is an empirical factor obtained by adding the total percentages of a sample of the aggregate retained on each of a specified series of sieves, and dividing the sum by 100. Additionally, the unit weight is the weight per unit volume of a material. It depends on the value of the gravitational acceleration. The density of a substance is its mass per unit volume [19,20,21].

**Figure 2:**
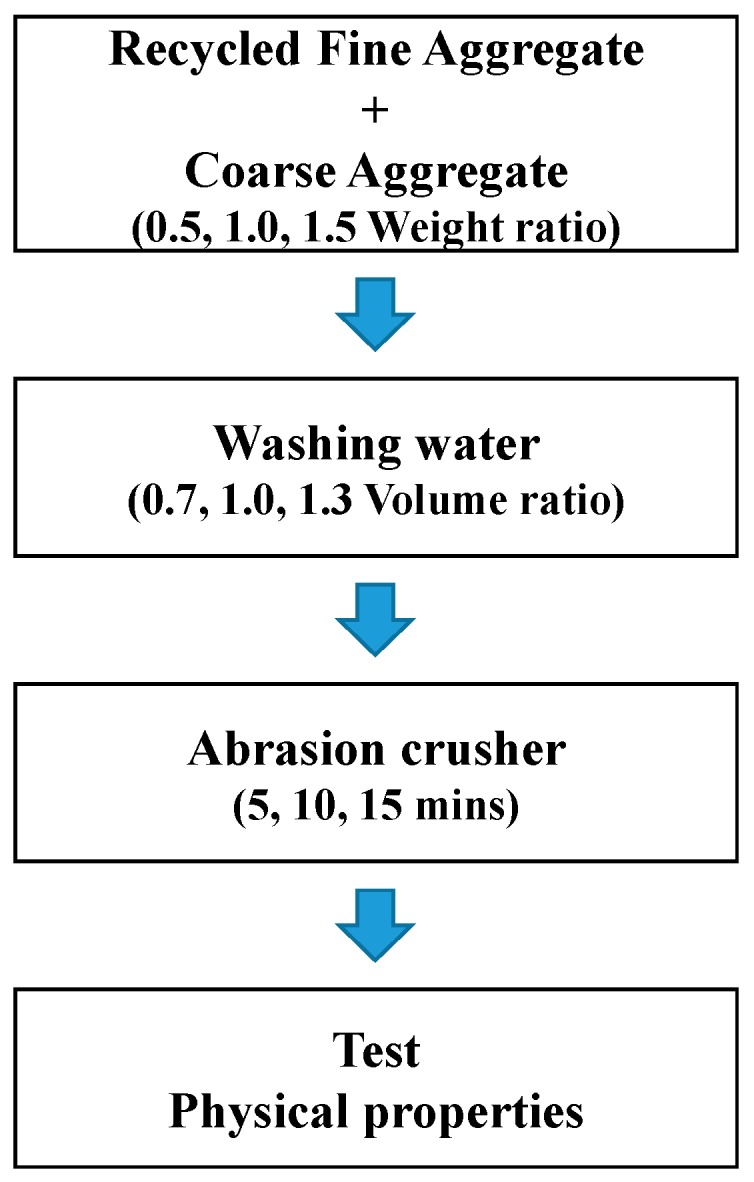
Experiment process of the manufacture of high-quality of recycled fine aggregate.

As shown in Figure 2, recycled fine and coarse aggregates were fed first into the system according to the weight ratio, and the washing water, made by diluting sulfuric acid, was fed into the system afterwards according to the volume ratio. Then, the experiment was conducted by varying the abrasion time. Furthermore, the tests on the density, absorption ratio and solid volume percentage of the recycled fine aggregates generated were conducted in accordance with KS F 2504 [22], which is similar to ASTM C128 [23], and KS F 2505 [24], similar to ASTM C29 [25], respectively.

### 2.3. Measurement Method

The testing on the quality of the recycled aggregates was conducted according to the items listed in the quality standards for recycled aggregates (limited to recycled fine aggregates used in concrete manufacture), and of the physical properties specified in the quality standards, the most important quality characteristics of recycled fine aggregates were reviewed and a statistical analysis was performed on the results. Table 4 shows the items that were measured in this study [22,24,26].

## 3. Results and Discussion

### 3.1. Analysis of the Results of the Experiment Performed According to an Orthogonal Design

Table 5 shows the results for the density, absorption ratio, and solid volume percentage according to the abrasion conditions that were obtained according to an orthogonal design.

#### 3.1.1. Oven-Dry Density

IBM SPSS Statistics (V19.0, IBM Corp., Armonk, NY, USA, 2015) [27], the commercial statistics software package, is selected to perform the F-test in this study. Table 6 shows the results of the F-test performed on each experimental parameter after performing ANOVA on the density results. The results of the ANOVA show that the F values of A (water ratio), B (coarse aggregate ratio), and C (abrasion time) were 3.0, 31.0, and 169.0, respectively. As for the experimental values, the degree of freedom was 2 and the error value was 2. Thus, the critical values, F_0.01_, F_0.05_, and F_0.10_, were found to be 99.0, 19.0, and 9.0, at a confidence limit of 99% (level of significance, α = 0.01), 95% (level of significance, α = 0.05), and 90% (level of significance, α = 0.10). Based on this, C (abrasion time) was found to satisfy the 99% (level of significance, α = 0.01) level, which means that it exerted the most significant impact on the quality of recycled aggregates. In addition, B (coarse aggregate ratio) satisfied the 95% (level of significance, α = 0.05) level, meaning that it had an impact on the quality. However, A (water ratio) was found to be insignificant even at a confidence limit of 90% (level of significance, α = 0.10), meaning that it did not exert any significant impact on quality.

**Figure 3:**
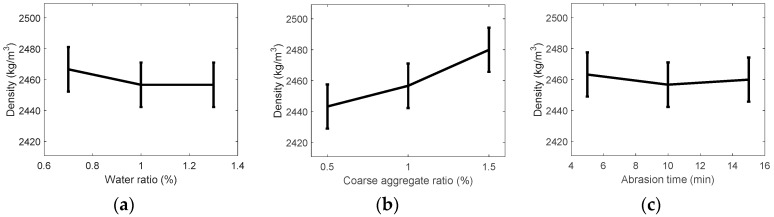
Assumed density of recycled fine aggregate by variance analysis: (**a**) water ratio; (**b**) coarse aggregate ratio; and (**c**) abrasion crusher time.

Figure 3 shows the density values estimated using the test results. The mean density varied between 2.24 and 2.51. As shown in the figure, coarse aggregate ratio and abrasion time were found to be positively correlated with density, and of particular note, abrasion time was found to be a major factor contributing to an increase in density. On the other hand, the water ratio, which was found to have little to no effect based on the F-test results, was shown to reduce the density, albeit slightly, if it was decreased.

#### 3.1.2. Absorption Ratio

Table 7 shows the results of the F-test performed on each experimental parameter after performing ANOVA on the absorption ratio results. The F values of A (water ratio), B (coarse aggregate ratio), and C (abrasion time) were found to be 6.50, 9.00, and 89.2, respectively. Since C (abrasion time) was larger than 19.0, the critical value at a confidence limit of 95% (level of significance, α = 0.05), it was determined that this parameter had the most significant impact on the post-abrasion absorption ratio. Additionally, B (coarse aggregate ratio) was found to be a significant factor at a confidence limit of 90% (level of significance, α = 0.10). In the case of water ratio, it was smaller than 9.0, the critical value at a confidence limit of 90% (level of significance, α = 0.10) and, thus, it was deemed to have little impact on the post-abrasion absorption ratio.

**Figure 4:**
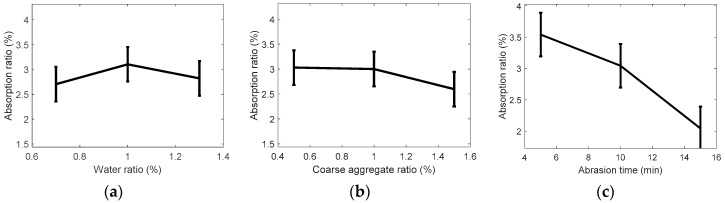
Assumed absorption ratio of recycled fine aggregate by variance analysis: (**a**) water ratio; (**b**) coarse aggregate ratio; and (**c**) abrasion crusher time.

Figure 4 shows the changes in the values estimated for the population mean for each level of the experimental parameters with respect to the absorption ratio. As shown in the figure, the absorption ratio decreased significantly with increased abrasion time. The absorption ratio also decreased with an increased coarse aggregate ratio, but not as substantially as the absorption ratio. On the other hand, the water ratio showed a point of inflection at 1.0, making it difficult to identify a consistent trend.

#### 3.1.3. Solid Volume Percentage

Table 8 shows the results of the F-test performed on each experimental parameter after performing ANOVA on the solid volume percentage results. The F values of A (water ratio), B (coarse aggregate ratio), and C (abrasion time) were found to be 0.60, 1.16, and 24.7, respectively. The sum of squares of A (water ratio) was smaller than the error term and, thus, A (water ratio) was included in the error for pooling.

Table 9 shows the pooling results. C (abrasion time) was higher than 18.0, the critical value at a confidence limit of 99% (level of significance, α = 0.01), while B (coarse aggregate ratio) was smaller than 4.32, the critical value at a confidence limit of 90% (level of significance, α = 0.10). Thus, C (abrasion time) was found as the most significant parameter even for the solid volume percentage, whereas A (water ratio) and B (coarse aggregate ratio) were found to be insignificant.

**Figure 5:**
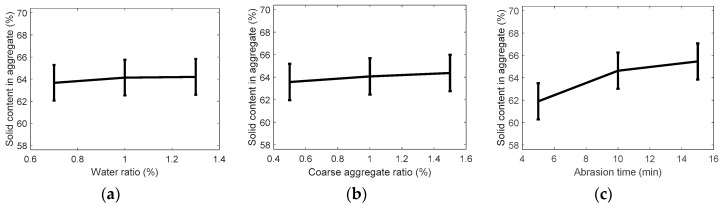
Assumed solid volume of recycled fine aggregate by variance analysis: (**a**) water ratio; (**b**) coarse aggregate ratio; and (**c**) abrasion crusher time.

Figure 5 shows the changes in the values estimated for the population mean for each level of the experimental parameters with respect to the solid volume percentage. Similar to the experimental results for density and absorption ratio, an increase in the abrasion time substantially increased the solid volume percentage. The increase was especially dramatic between 5 and 10 min of abrasion time. On the other hand, an increase in the water ratio or the coarse aggregate ratio caused only a slight increase in the solid volume percentage. An increase of processing time in abrasion crusher results in an increase of friction between the recycled fine aggregates. Therefore, it leads to accelerating the removal of cement paste from the recycled fine aggregates, enhancing the grain size of aggregates, etc. Finally, these actions improve the solid contents in aggregates, which is shown in Figure 5c.

### 3.2. Response Surface Methodology and Optimization

RSM was applied with the aim to determine the abrasion conditions that would induce optimum performance based on the results derived by applying an orthogonal array design. The target performance levels were over 2460 oven-dry density and less than 3.0 absorption ratio. It should be noted that the recycled fine aggregate, which meets the quality standard, has been limited to use in concrete manufacture. To overcome it, the quality standard for the natural fine aggregate, which is over 2450 density and less than 3.0 absorption ratio, has been considered as the quality standard for the recycled fine aggregate in this study. The solid volume percentage was omitted as it was shown to be excellent in all experimental values at over 60%. Moreover, as analyzed above, A (water ratio) was excluded as it was determined to have low significance, and RSM was applied only for B (coarse aggregate ratio) and C (abrasion time). The results were then compiled to derive the optimum abrasion conditions.

#### 3.2.1. Density

Relationship between two factors, i.e., the coarse aggregate ratio and the abrasion time, and the density of recycled fine aggregate was derived by using Equation (3) and can be expressed as:

(3) $$Y_{D}=2.3833-0.0333\times x_{1}+0.0057\times x_{2}+0.02\times x_{1}^{2}+0.003\times x_{1}\times x_{2}$$

where *Y_D_* is the density of recycled fine aggregate, *x*_1_ is the coarse aggregate ratio, and *x*_2_ is the abrasion time.

Table 10 shows the results of applying RSM with respect to density in cases where there were changes in B (coarse aggregate ratio) and C (abrasion time). R2, the coefficient of determination for the regression equation, with respect to density, was 0.99, and its significance within the significance level of 99% was recognized.

**Figure 6:**
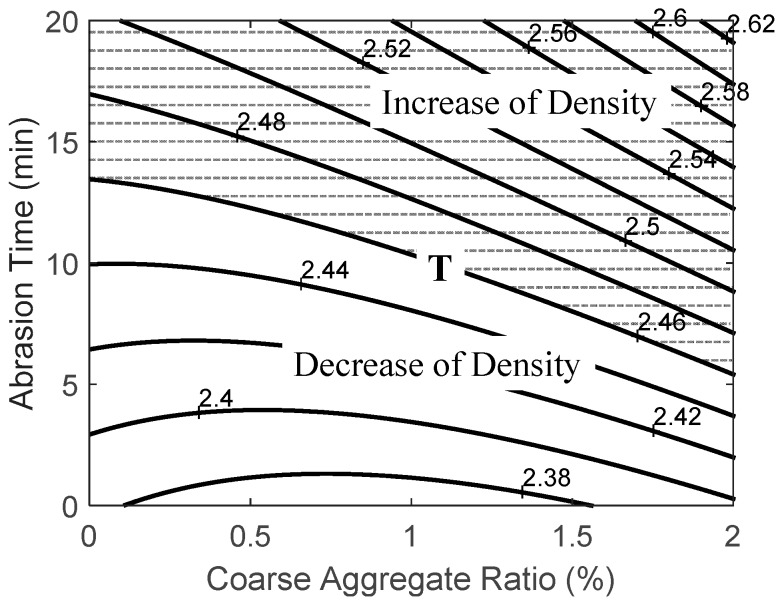
Analysis response surface for the density of recycled fine aggregate drive from coarse aggregate ratio and abrasion time.

Figure 6 shows the results of applying RSM in relation to the oven-dry density of recycled fine aggregates that were obtained from B (coarse aggregate ratio) and C (abrasion time). Line T in Figure 6 is a line that satisfies the target performance level for the oven-dry density, which was 2.46. In the case of recycled fine aggregates, increased density results in quality improvement and, thus, the upper part of Line T satisfies the target performance level. The target performance level was satisfied at an abrasion time of over 11 min for a coarse aggregate ratio of 1.0, over 8 min for a coarse aggregate ratio of 1.5, and over 6 min for a coarse aggregate ratio of 2.0. Increased abrasion time means increased input energy, and this improves the quality of recycled aggregates. Increasing the coarse aggregate ratio, on the other hand, results in a relative decrease in the target amount of recycled fine aggregates as the capacity of the abrasion device is fixed. Therefore, these variables should be considered to determine the optimum coarse aggregate ratio and abrasion time.

#### 3.2.2. Absorption Ratio

Again, the relationship between two factors, i.e., the coarse aggregate ratio and the abrasion time, and the absorption ratio of recycled fine aggregate was derived by using Equation (4) and can be expressed as:

(4) $$Y_{AR}=2.7244+1.68\times x_{1}+0.1137\times x_{2}-0.7467\times x_{1}^{2}-0.062\times x_{1}\times x_{2}-0.0101\times x_{2}^{2}$$

where *Y_AR_* is the absorption ratio of recycled fine aggregate, *x*_1_ is the coarse aggregate ratio, and *x*_2_ is the abrasion time.

Table 11 shows the results of applying RSM with respect to the absorption ratio in cases where there were changes in B (coarse aggregate ratio) and C (abrasion time). R2, the coefficient of determination for the regression equation with respect to absorption ratio, was 0.95, and its significance within the significance level of 90% was recognized.

**Figure 7:**
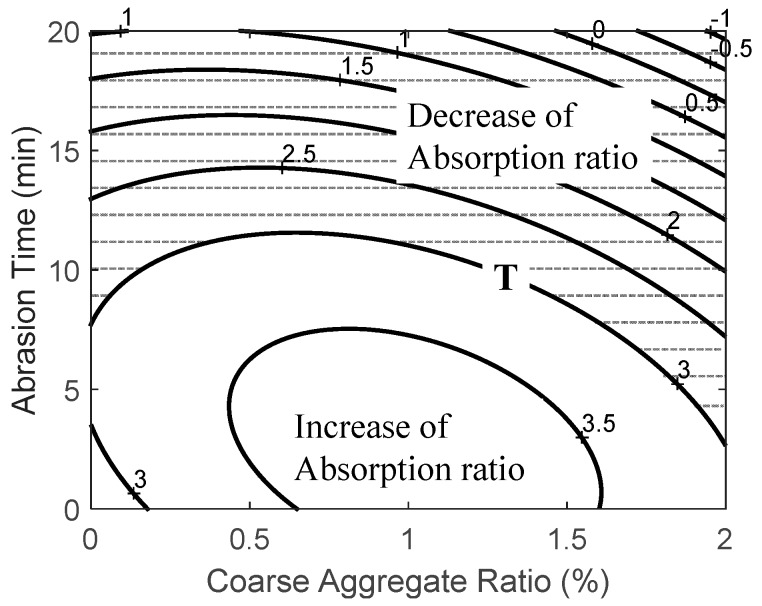
Analysis response surface for the absorption ratio of recycled fine aggregate derived from the coarse aggregate ratio and abrasion time.

Figure 7 shows the results of the contour analysis of the optimum abrasion range for the absorption ratio according to B (coarse aggregate ratio) and C (abrasion time). The results show that, similar to density, an abrasion time of over 15 min and fine aggregate and coarse aggregate ratio of over 1 resulted in a performance level exceeding the target level proposed in the quality standards for recycled fine aggregates, which is up to the standard in comparison to natural fine aggregates.

#### 3.2.3. Derivation Optimum Abrasion Conditions

An optimization technique is applied to seek the optimum abrasion conditions. Typically, an optimization problem with constraints can be expressed as:

*Maximize f(x)* (5)

(6) $$\text{Subjected to}\;\{\begin{array}{ccc} g_{i}\left(x\right)\leq 0 & : & i=1,2,\ldots ,q\\ h_{j}\left(x\right)=0 & : & j=q+1,q+2,\ldots m \end{array}$$

where *f(x)* is an objective function, *g(x)* are the inequality constraints, *q* is the number of inequality constraints, *h(x)* are the equality constraints, and *m*–*q* provides the number of equality constraints [28].

It is difficult to determine the global maximum or minimum solution if the objective function does not have one or more constraints. Therefore, a penalized objective function is adopted and is expressed as follows [28]:

(7) $$f_{p}\left(x\right)=f\left(x\right)+\overset{m}{\underset{i=1}{\sum }}C_{i}{\left(P_{i}\right)}^{\beta }$$

(8) $${\left(P_{i}\right)}^{\beta }=\{\begin{array}{ccc} \delta_{i}g_{i}\left(x\right) & : & i=1,2,\ldots ,q\\ \left|h_{j}\left(x\right)\right| & : & j=q+1,q+2,\ldots m \end{array}$$

(9) $$\delta_{i}=\{\begin{array}{ccc} 1 & : & \text{if}\;i-th\;\text{constraint is violated}\;(\mathrm{i.e.},\;g_{i}\left(x\right)>0)\\ 0 & : & \text{otherwise}\;\left(\mathrm{i.e.},\;{\text{g}}_{i}\left(x\right)\leq 0\right) \end{array}$$

where *f_P_(x)* is a penalized objective function, *f(x)* is the (unpenalized) objective function, *C_i_* is a value imposed for violation of the *ith* constraint with values equal to a relatively large number, β is a user-defined exponent, with values of 1 or 2 typically used, δ*_i_* is the Kronecker delta function, and constraints 1 through *q* are inequality constraints. One can see that the penalty will only be activated when the constraint is violated, while constraints *q* + 1 through m are equality constraints that will activate the penalty if there are any non-zero values [28,29].

In this study, the productivity per day, i.e., the unit productivity, is introduced as the objective function and is expressed as a function of abrasion time and coarse aggregate ratio. It should be noted that the actual equation for the unit productivity is very complicated and must consider various factors, such as human labor, material cost, delivery fee, machine operations time, etc. Rather, it is simply assumed like a unit time to product, which is 8 h per day divided by the abrasion time, multiplying the amount of coarse aggregates, and is expressed as:

(10) $$Q_{day}=\frac{480}{x_{2}}\times \frac{x_{1}}{2}$$

where *Q_day_* is an expression for the unit productivity, *x*_1_ is coarse aggregate ratio, and *x*_2_ is abrasion time.

Design variables, i.e., *x*_1_ is coarse aggregate time and *x*_2_ is abrasion time, are selected and have a range from 0.5% to 1.5% and from 5 min to 15 min, respectively. To determine optimal conditions, six constraints are selected: (1) lower/upper limits for coarse aggregate ratio and abrasion time; (2) lower/upper limits for density; and (3) lower/upper limits for absorption time. Density and absorption time are derived and are expressed by means of design variables in Table 10 and Table 11, respectively. Assumptions for density and absorption time are introduced for the optimization problem in this study, i.e., the aforementioned target performance levels are considered as the limitations for density and absorption time. In other words, density should be greater than the target performance level, i.e., 2.46. Absorption time should be less than the target performance level, i.e., 3.0, and should be greater than zero. Thus, constraints are expressed as:

*x*_1_ Limits:

(11) $$g_{1}\left(x\right)=0.5\leq x_{1}=1-\frac{x_{1}}{0.5}\leq 0$$

(12) $$g_{2}\left(x\right)=x_{1}\leq 1.5=1-\frac{1.5}{x_{1}}\leq 0$$

*x*_2_ Limits:

(13) $$g_{3}\left(x\right)=0.5\leq x_{2}=1-\frac{x_{2}}{5}\leq 0$$

(14) $$g_{4}\left(x\right)=x_{2}\leq 15=1-\frac{15}{x_{2}}\leq 0$$

Density Limits:

(15) $$g_{5}\left(x\right)=2.46\leq Y_{D}=1-\frac{Y_{D}}{2.46}\leq 0$$

Absorption Limits:

(16) $$g_{6}\left(x\right)=Y_{AR}\leq 3=1-\frac{3}{Y_{AR}}\leq 0$$

where *x*_1_ is the coarse aggregate ratio, *x*_2_ is the abrasion time, *Y_D_* is the density, and *Y_AR_* is the absorption time.

**Figure 8:**
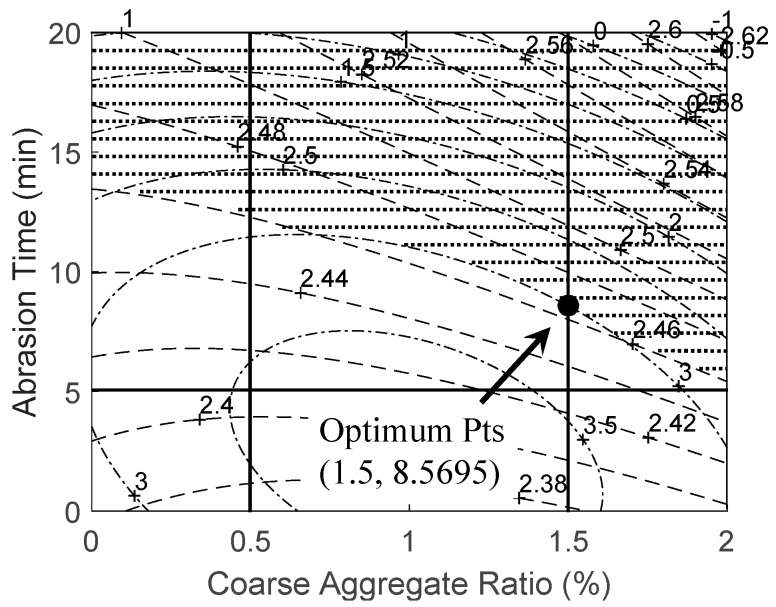
The optimum condition for improved quality of recycled fine aggregate.

Optimization has been performed using MATLAB [30] and Figure 8 shows the optimum mixing conditions satisfying the required performance level that was determined based on Figure 6 and Figure 7, showing the results of analyzing the density and the absorption ratio. The results of the contour analysis showed that aggregates manufactured through a process had an oven-dry density level of over 2200 kg/m^3^ and absorption ratio of under 5%, which are the standard quality levels prescribed in the quality standards for recycled aggregates, and satisfied the standards for natural aggregates proposed in KS, which are a density of over 2460 kg/m^3^ and an absorption ratio of under 3%. Thus, considering the productivity of the manufacture of recycled fine aggregates, it is deemed that it would be economical to feed relatively large amounts of coarse aggregates to reduce the production time. In addition, the optimum abrasion conditions for improved productivity and cost effectiveness would be an abrasion time of 8.57 min, i.e., 8.5695 min is the exact value, and a coarse aggregate ratio of over 1.5. In that case, the unit productivity was maximized and has a value of 42.01.

## 4. Conclusions

Typically, the recycled aggregates have a limited usage due to its low quality comparing with natural aggregates. In this study, experimental testing has been conducted considering three factors, i.e., the fine aggregate to water ratio, the fine aggregate to coarse aggregate ratio, and the abrasion time, to improve the quality of the recycled aggregates. To do this, experimental plans have been arranged in a three-level system using the orthogonal design method. Overall, 27 experimental plans have been arranged in nine experimental levels. The abrasion conditions for the production of recycled fine aggregates have been derived using the response surface methods and its optimum conditions to maximize the production have been found using the Nelder-Mead sequential simplex algorithm with various constraints. During the procedure, the unit production amount has been selected as the objective function.

The following conclusions were made based on the results of this study conducted on the manufacturing method for recycled fine aggregates that satisfy the quality standards. Of the abrasion conditions, such as water ratio, coarse aggregate ratio, and abrasion time, abrasion time was found to have the most significant impact on the density change, while the water ratio had no significant impact. The results of reviewing the changes in the quality of recycled fine aggregates caused by varying the water ratio, coarse aggregate ratio, and abrasion time showed that, similar to the density experiment, abrasion time was found to have the most significant impact on the changes in the absorption ratio, and the trend in the changes was similar to that of the changes observed in density. The results of the abrasion experiment performed to determine the optimum conditions for the manufacture of recycled fine aggregates that satisfy the quality standards showed that an abrasion time of 8.57 min and coarse aggregate ratio of over 1.5 have been the optimum conditions for producing aggregates with the target oven-dry density of 2460 kg/m^3^ and absorption ratio of under 3%.

Therefore, it is advantageous to use pulverized materials to ensure the quality of the production efficiency and aggregate when the optimal abrasion conditions for producing the high-quality of the recycled aggregates are applied. Additionally, it gives more benefits in productivity and economics to use the recycled aggregates from deconstruction and demolition of reinforced concrete structures and will be helpful to save the limited amount of the natural aggregates and environmental protection. Finally, it can be expected that a further study related to apply the recycled aggregates, having the improved quality, to the mortar and concrete in order to evaluate its material properties and stability.

## Figures and Tables

**Table 1:** Experimental plan using the design of experiment.

Factors		Levels			Test Items
		0	1	2	
A	Water ratio ^1^ (%)	0.7	1.0	1.3	Density
B	Coarse aggregate ratio ^2^ (%)	0.5	1.0	1.5	Absorption ratio
C	Abrasion time (min)	5	10	15	Percentage of solid volume

^1^ Volume ratio of water to total aggregate; ^2^ Weight ratio of coarse aggregate to fine aggregate.

**Table 2:** The level of experiment.

ID	A	B	C	Levels ^1^
	Water Ratio (%)	Coarse Aggregate Ratio (%)	Abrasion Time (min)	
A_0_B_0_C_0_	0.7	0.5	5	A_0_ = 0.7
A_1_B_0_C_2_	1.0	0.5	15	A_1_ = 1.0
A_2_B_0_C_1_	1.3	0.5	10	A_2_ = 1.3
A_0_B_1_C_1_	0.7	1.0	10	B_0_ = 0.5
A_1_B_1_C_0_	1.0	1.0	5	B_1_ = 1.0
A_2_B_1_C_2_	1.3	1.0	15	B_2_ = 1.5
A_0_B_2_C_2_	0.7	1.5	15	C_0_ = 5
A_1_B_2_C_1_	1.0	1.5	10	C_1_ = 10
A_2_B_2_C_0_	1.3	1.5	5	C_2_ = 15

^1^ 0, 1, and 2 represent the experimental level, respectively.

**Table 3:** The physical properties of recycled fine aggregate.

Density (kg/m^3^)	Absorption Ratio (%)	Fineness Modulus	Solid Content in Aggregate (%)	Unit Weight (kg/m^3^)
2270	6.56	3.40	59.6	1443

**Table 4:** Testing items and their measurement methods.

Item	Experimental Method	Standard of Recycled Fine Aggregate
Density (kg/m^3^)	KS F 2503 (ASTM C128)	2.2 over
Absorption ratio (%)	KS F 2504 (ASTM C128)	5.0 under
Solid content in aggregate (%)	KS F 2505 (ASTM C29)	53 over

**Table 5:** Test results.

ID	Density (kg/m^3^)		Absorption Ratio (%)	Solid Content in Aggregate (%)
	Oven-Dry	Saturated Surface-Dry		
Base	2270	2420	6.56	59.6
A_0_B_0_C_0_	2410	2500	3.43	60.7
A_1_B_0_C_2_	2480	2540	2.46	65.4
A_2_B_0_C_1_	2440	2520	3.20	64.6
A_0_B_1_C_1_	2460	2540	3.03	64.6
A_1_B_1_C_0_	2410	2500	3.95	62.3
A_2_B_1_C_2_	2500	2550	2.02	65.3
A_0_B_2_C_2_	2530	2570	1.65	65.7
A_1_B_2_C_1_	2480	2550	2.90	64.7
A_2_B_2_C_0_	2430	2500	3.24	62.7

**Table 6:** Variance analysis for density of recycled fine aggregate.

ID	Factors	S ^1^	Ø ^2^	V ^3^	F_0_^4^	Evaluation
A	Washing water	0.0002	2	0.0001	3.00	-
B	Coarse aggregate	0.0021	2	0.0010	31.00	**
C	Abrasion time	0.0113	2	0.0056	169.00	***
Error		0.0001	2	0.0000		
Total		0.0136	8			

^1^ S: sum of squares; ^2^ Ø: degree of freedom; ^3^ V: mean of the sum of squares; ^4^ F_0_: F-statistics value; *** accepted at the 0.01 significance level; ** accepted at the 0.05 significance level; - not accepted.

**Table 7:** Variance analysis for absorption ratio of recycled fine aggregate.

ID	Factors	S	Ø	V	F_0_	Evaluation
A	Washing water	0.2539	2	0.1269	6.4952	-
B	Coarse aggregate	0.3514	2	0.1757	8.9886	*
C	Abrasion time	3.4867	2	1.7433	89.1990	***
Error		0.0391	2	0.0195		
Total		4.1310	8			

*** accepted at the 0.01 significance level; * accepted at the 0.10 significance level; - not accepted.

**Table 8:** Variance analysis for the solid volume of recycled fine aggregate.

ID	Factors	S	Ø	V	F_0_	Evaluation
A	Washing water	0.5067	2	0.2533	0.5984	-
B	Coarse aggregate	0.9800	2	0.4900	1.1575	-
C	Abrasion time	20.8867	2	10.4433	24.6693	***
Error		0.8467	2	0.4233		
Total		23.2200	8			

*** accepted at the 0.01 significance level; - not accepted.

**Table 9:** Variance analysis for the solid volume of recycled fine aggregate after pooling.

ID	Factors	S	Ø	V	F_0_	Evaluation
B	Coarse aggregate	0.9800	2	0.4900	1.45	-
C	Abrasion time	20.887	2	10.443	30.9	***
Error		1.3534	4	0.3384		
Total			8			

*** accepted at the 0.01 significance level; - not accepted.

**Table 10:** Variance analysis for the density of recycled fine aggregate.

Factors	S	Ø	V	F_0_	Evaluation
Regression	0.0136	5	0.0027	195.2400	***
Error	0.0000	3	0.0000		
Total	0.0136	8			

*** accepted at the 0.01 significance level.

**Table 11:** Variance analysis for absorption ratio of recycled fine aggregate.

Factors	S	Ø	V	F_0_	Evaluation
Regression	3.9341	5	0.7868	11.9896	**
Error	0.1969	3	0.0656		
Total	4.1310	8			

** accepted at the 0.05 significance level.

## Abbreviations

The following abbreviations are used in this manuscript:

S	Sum of squares
Ø	Degree of freedom
V0	Mean of the sum of squares
F_0_	F-statistics value
RSM	Response surface methodology
